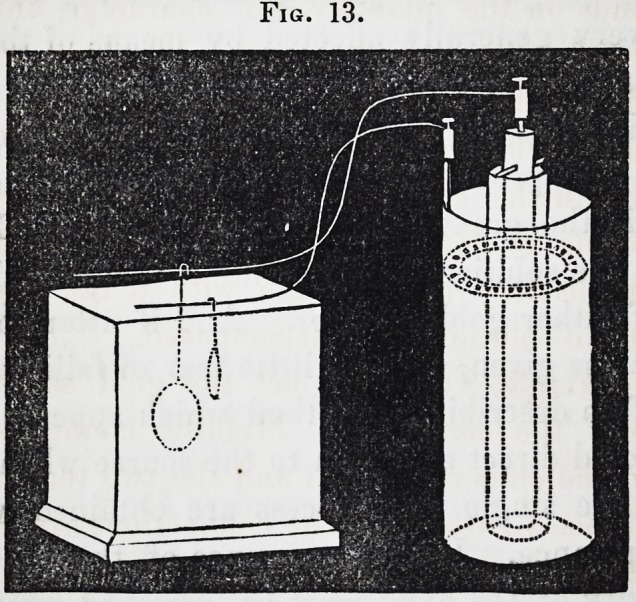# Electrotype Manipulation.—Part II

**Published:** 1856-01

**Authors:** 


### ARTICLE XVII.

### *Electrotype Manipulation.—Part II.*

*I.—Introductory Observations.*—92. HAVING in Part I given the mode of working in copper, we now pass to other metals. First in importance come gold and silver. The earliest experiments in electro-gilding were those by Brugnatelli, who gilded silver medals by electricity, in 1805; he used a solution of nitro-muriate of gold, mixed with a solution of ammonia. The next were those of De la Rive, 1841, who used a solution of chloride of gold. But these processes were interesting rather in a theoretrical than in a practical point of view; inasmuch as the elective chemical affinity of the elements (combined in these solutions with the gold) for the baser metals, which might be immersed in the solutions, is such, that a violent interchange of elements takes place, and the gold is set free without even electric agency; and the solutions are so readily decomposed by the smallest adventitious aid, that it is a practical impossibility to obtain a "reguline" deposit, however much the voltaic power may be modified.

The following are illustrations of the deposition of gold and silver by the mere elective affinity or ordinary chemical action. If an aqueous solution of chloride of gold is agitated with ether, the chlorine leaves the water to combine with the ether, and the resulting compound, being lighter than water, floats on the surface.—If pieces of polished steel are dipped into this preparation they acquire a coat of gold by ordinary chemical interchange.—If a design is traced with solution of chloride of gold upon a silk or linen fabric, and the fabric, while the traces are still moist, be exposed to a stream of hydrogen gas

(which may readily be obtained by acting upon iron nails by diluted sulphuric acid,) the metal is reduced, and a golden design is the result.—If a plaster cast is saturated with a solution of nitrate of silver, and placed under a bell-glass, and we admit to it the gas produced by heating a few grains of phosphorus with alcohol and a small quantity of potash, the silver will be reduced upon the surface.—If the plaster-cast is made with sour whey instead of water, and is saturated with silver solution, the silver is reduced by mere exposure to sunlight, and forms, according to Elsren, a good conducting surface, in which we can deposit copper or silver according to the usual modes.

The same observations apply to the ordinary salts of silver, as, for example, the nitrate, &c. In fact, long before the theory of chemical deposits was understood, I made some experiments upon the electrolysis of this salt, and succeeded in producing an electrotype medal with a *silver* surface, being I think the *first* instance of electro-plating, when the *object of the experiment was electro-plating.* But I was unsuccessful in my attempts to repeat the experiments; and simply because, in that instance, I *chanced* to have in action a power nicely *balanced* with the work to be performed, but in future instances my power was not adjusted to the work.

93. The first practical process for working in these noble metals is undoubtedly due to the patentees, Messrs. Elkington. Others have laid claim to having been the first to use solutions similar to theirs; but whatever may have been done by these others in private, it does not appear that the public were in possession of their processes by any authentic publication; and therefore there is no alternative but to give the patentees the claim of originality and priority.

94. The solutions they employ are the *argento-cyanide* and the *auro-cyanide* of potassium; upon which compounds it will be well if we make a few observations here, at the outset. They are what the chemist term double salts: as, for instance, *cyanide of potassium* is a compound simply of potassium and cyanogen; *argento-cyanide of potassium* is silver and cyanogen combined with potassium and cyanogen, or, which amounts to the same thing, cyanide of silver united with cyanide of potassium.

When viewing (§ 13) what happened during the decomposition of sulphate of copper, we had occasion to describe that body as *oxyd of copper*, dissolved in, or combined with, *sulphuric acid:* now, oxyd of copper is 1 part copper + 1 part oxygen, and sulphuric acid is 1 part sulphur + 3 parts oxygen, and a certain quantity of water; so that, altogether, the arrangement is somewhat complex. This is not so much the case with the bodies now in question. And first, in respect to the simple *cyanide of potassium*, before it is united with the gold or silver. It consists simply of one equivalent of the metal potassium, and one equivalent of cyanogen; and, when it is acted upon by a voltaic current in the usual way, it appears to be decomposed by *direct* action, and cyanogen is liberated at the one pole, and potassium is *determined* to the other, but not *liberated.* It will be remembered (§ 13) that, in the solution of sulphate of copper, *water* was decomposed by the *direct* action, and that the copper was liberated by a *secondary* action, namely, by the hydrogen of the water returning back into solution in the place of the copper. Well; the cyanide of potassium is decomposed by *direct* action, and potassium is presented to the negative metal; but a *secondary* action now occurs: so great is the affinity of potassium for oxygen, that it cannot exist in a metallic form in presence of that element; as is well known from the common experiment of dropping a piece of potassium upon water, when it combines so violently with the oxygen as to produce heat and light; and the resulting products are oxyd of potassium, the common *caustic potash*, accompanied with a liberation of hydrogen. So, also, in the present case; the potassium does not itself appear, but, in its place we find hydrogen and potash; it takes oxygen from the water and forms potash, and sets the hydrogen free. It is true, we are in possession of a means of preventing its return into solution, and this is by employing a mass of mercury to receive it: in which case it unites with the mercury and forms the amalgam of potassium; and neither hydrogen nor potash is manifested; but it will remain thus only under favorable circumstances; for, if the connections with the battery are broken, so that the mercury ceases to be negatively electrified, the potassium immediately leaves it, and decomposes the water as before. Thus much in reference to the simple cyanide of potassium. Of the double cyanides, the argento may be taken as an example. It consists of 1 part cyanide of potassium and 1 part cyanide of silver—the latter cyanide, like the former, consisting of 1 part metal+1 cyanogen. When a solution of this double cyanide is electrolized, silver appears at one pole, and cyanogen at the other. But in order to the production of this result, it is absolutely essential that there be a considerable surplus quantity of the cyanide of potassium in solution; indeed, it is pretty evident that the direct action is the decomposition of the surplus cyanide, and that the silver is reduced by secondary action in the following way. When the metal potassium is reduced from its cyanide, it returns into solution, and takes the place of the silver in the double salt, setting the latter metal free; so that, while on the one hand an equivalent of simple cyanide is consumed, on the other hand an equivalent is formed, and the equivalent previously engaged to form with the silver the double salt, is also free; and thus far there is an increase in the quantity of simple cyanide of potassium. But, if the positive metal is silver, the cyanogen combines with it and forms cyanide of silver; for cyanogen is a gas, and like oxygen seems to combine with metals in this its nascent state; though, unlike oxygen, it is a compound body, consisting of two equivalents of carbon + 1 of nitrogen, whence it is also termed bicarburet of nitrogen. Well; cyanide of silver is insoluble in water, and hence would form an insulating crust on the silver plate were it not for the presence of cyanide of potassium in excess in solution; it readily dissolves in this, and so keeps up the strength of the solution and the extra element of cyanide of potassium, mentioned above, is thus neutralized.

Having thus described the general character of the cyanide solution, it remains for us to give the processes by which the several elements are most favorably brought together.

95. *Cyanide of Potassium.*—To obtain this, we set out with the ferro-cyanuret of potassium, or yellow prussiate of potash of commerce; and as this prussiate is readily accessible at all chemists, it is better in general to purchase than to make it; the mode by which it is obtained will be found in any treatise on chemistry. It consists of 1 equivalent of cyanide of iron + 2 equivalents of cyanide of potassium. It is of a bright yellow color, and is converted into the colorless simple cyanuret in the following manner. Take 4 oz. of the yellow prussiate, break it in small pieces, and dry it well on a plate of iron; then reduce it in a mortar to exceedingly fine powder. Dry and pound in like manner $1\frac{1}{2}$ oz. of carbonate of potash. Incorporate the two ingredients thoroughly. Place a Hessian crucible in the fire; and when it attains a red heat, throw into it the prepared mixture, and closely cover the crucible. Keep up the heat, and the contents of the curcible will soon fuse, and the fluid mass will become redhot. After this, immerse in it, from time to time, a hot glass rod; the mass that adheres to the rod in the early stages of the process is brown on cooling; as the heat is continued, it appears yellowish, and finally colorless and transparent. The operation is then complete; the crucible must be removed; and after its contents have been allowed to settle, the fused mass may be poured off; the greater portion of which consists of the *simple cyanuret of potassium.** The impurities contained in this product are not detrimental to its use, in a general way, for the purposes in view; however, in cases where it is required pure, it must be boiled in strong alcohol; and when the alcohol cools, the pure cyanide will be deposited in the form of small white crystals. This salt is very deliquescent, and must therefore be retained in close bottles; it will readily be recognized by its powerful odor—similar to that produced by peach-blossoms. The mere mention of *prussic acid* almost entering into its composition will be sufficient to induce my readers to exercise common caution in handling it.—A solvent solution is prepared by adding two ounces of this salt to a pint of rain or distilled water; when the salt is well dissolved, the liquid is ready for use.

* This method was first described by Messrs. Rodgers, in the *Philosophical Magazine* for Feb. 1834; and since by Prof. Liebig.

96. *Silver Solution.*—Silver may be presented to the above solution in various forms; as the oxyd, the chloride, the carbonate, the nitrate, &c.; solution will in either case occur; and the double cyanide of silver and potassium will be produced. But since the silver, as we hinted before, must become a cyanide of silver before it can thus unite with the cyanide of potassium, it is obvious that one portion of the solution must give up its cyanogen to the silver, and take to itself the bodies previously in combination with that metal. So that, from the oxyd of silver, potash would occur in the solution; from chloride, chloride of potassa; from carbonate, carbonate of potassa; and from nitrate, saltpetre. Of these the least likely to interfere with this general action is the potash; and hence oxyd of silver has been frequently used. It is thus prepared:—

97. *Oxyd of Silver.*—Place pieces of silver in a glass vessel, and pour on them about equal parts of water and strong nitric acid; the metal will soon dissolve, giving off fumes of nitric oxyd. Should the solution have a green hue, which is invariably the case unless the metal has been obtained fine from the refiners, it indicates the presence of copper; in which case immerse some pieces of copper in the solution, and the nitric acid, by elective affinity, will combine with the copper; and a precipitate of pure silver, in the form of a grayish powder, will take place. Throw away the liquid, and wash the silver precipitate several times in sulphuric acid and water, and afterwards in water alone. Then redissolve it, as before, in nitric acid and water; and a *solution of pure nitrate of silver* will be obtained. Place this in an evaporating dish, or a saucer, and apply the heat of a spirit-lamp, or place the saucer by the fireside, till some portion of the liquid is driven off in vapor. Allow the residue to cool, and it will shoot out into long, colorless, transparent crystals which are *nitrate of silver.* They must be handled with care, as they possess the property of staining animal and vegetable substances with an almost indelible black; fused nitrate of silver being the lunar caustic of surgery, aud the main ingredient also of marking-ink.—Next prepare some *lime-water*, by stirring lime into water, and filtering the solution. As lime is very sparingly soluble in water, requiring at 60° Fahrenheit 750 times its weight, it is necessary to make an abundant supply. Place the lime-water in a glass or other vessel, and drop in it a *few* crystals of nitrate of silver: the colorless solution will instantly assume an unsightly brown hue; and, after remaining quiescent for a time, the *oxyd of silver* will subside in the form of a dark brown precipitate. The liquid is then poured off, and the precipitate is washed with water. Before throwing away the liquid, fresh lime-water should be added to it; and if the dark hue recurs, the precipitate must be allowed to subside again: if no change takes place, it may be inferred that the silver is all extracted. The oxyd of silver should not be dried, but be kept in bottles with water. A quarter of an ounce of oxyd of silver, added to a pint of the solvent solution, forms a very useful plating solution.

98. *Cyanide of Silver.*—But, as the above solution is impure, in that it contains as much potash as is equivalent to the oxyd of silver added, it may not be applicable to accurate experiments; and as the potash is produced, in the formation of cyanide of silver, at the expense of a certain portion of the cyanide of potassium, it is a wise plan, for it is no more costly, to form the cyanide of silver in a separate vessel, and to wash away the impurities before adding it to the solvent. Take then a neutral solution of nitrate of silver; add carefully a solution of cyanide of potassium, when a white precipitate of cyanide of silver will fall; continue adding until precipitation ceases. The liquid, which is a solution of nitrate of potash or saltpetre, is to be poured off, and the precipitate well washed. It will be pure cyanide of silver, if the materials employed were pure; and it is now fit to be added to the solvent liquid, to form a plating solution free from impurities.

99. *Preparation of the Gold Solution.*—Warm a pint of pure rain or distilled water, and dissolve in it two ounces of cyanide of potassium as before; then add a quarter of an ounce of oxyd of gold. The solution will at first be yellowish, but will soon subside to colorless transparency. Those not versed in chemical manipulation will be wiser to purchase than to prepare oxyd of gold; but, for general information, I give the process. Dissolve pure gold in two measures of muriatic with one of nitric acid; evaporate to dryness; dissolve the residuum in twelve times its weight of water; add to this a solution of pure carbonate of potash, dissolved in twice its weight of water; apply a moderate heat, about 170°, and a reddish-yellow precipitate occurs. This is the *hydrated* peroxyd of gold. Wash it well; and, to render it anhydrous, boil it in water. It then assumes a brownish-black color, which is the oxyd required.

100. I by no means give these as *standard* proportions of the several ingredients required. They are the proportions which I employed with success in gilding and plating the series of metals (submitted to the Electrical Society at their meeting, Sept. 21, 1841,) by the battery process to be hereafter described. When the same object is effected by the employment of a single cell, it will be requisite to alter the degree of saturation according to circumstances; to which, however, I shall have further to allude in the sequel.

101. *Single Cell for Plating and Gilding.*—The necessity of economizing solutions of such value as these has led to certain modifications in the apparatus contributing to that end. The porous cell (§ 17,) which in other arrangements contains the *zinc* and acid, and is surrounded by the copper or other negative element, in the present process contains the *cyanide solution*, and the negative element or object to receive the deposit, and is surrounded by the zinc, &c.

Fig. 11.

102. This arrangement will be readily understood by a glance at the annexed wood-cut, which represents a porcelain cell containing a cylinder of zinc, and an inner porous tube filled with the solution of silver or gold. Connection is made between the zinc and medal or mould by a binding screw; or by a mere contact, as in the figure.

103. I must again dwell upon the philosophy of the action of this arrangement, and return to first principles, in order to impress them more firmly on the minds of those who read these pages with the intent to repeat the experiments. For it is a matter of some importance, in employing the costly salts of the noble metals, to have the principles of the experiment traced out as distinctly as possible.

104. In the arrangement just described, the nature of the deposit will depend upon the principles elsewhere (§ 78) set forth; and *à fortiori*, from the facility with which the salts of silver or gold are decomposed, there will be a much *greater* chance of releasing hydrogen, and spoiling the experiment; to prevent which, therefore, ample provision must be made. For instance, if the silver solution is *weak* in proportion to the *energy* of action between the zinc and acid water, the electricity developed will be *more* than sufficient to release pure metal, and hydrogen will be evolved, the result being a deposition of oxyd. Or, if the balance between the strength of the solutions be duly adjusted, the relation between the size of the zinc and of the medal or mould may be such as to determine the same result. It is therefore requisite that the water which excites the zinc should contain *very little acid*—a few drops, more or less in proportion as the cyanide solution contains more or less of the oxyd; and that the strength of the latter should be maintained by a fresh supply of oxyd from time to time.

Fig. 12.

105. Another, and in some cases more convenient form for the single-cell apparatus is given in the annexed woodcut; in principle it differs from the former; the porous cell to contain the cyanide solution being flat, affords the means of immersing a larger medal, without an extravagant supply of liquid. The zinc which envelops the porous cell is also flat. The connections are made as before.

106. *Plating by means of a single Cell.*—Having charged either of these arrangements with the weak acid water and the solution of silver, let it remain for a few minutes, in order that the porous cell may be moistened through, and that action may commence as soon as the circuit is completed. Then attach a *thin,** pliable wire to the medal or mould, and place its other end in contact with the wire attached to the zinc: complete the circuit by immersing the metal in the silver solution, and a disposition will *instantly* take place. It will present a *dead* whitish appearance. At the meeting of the British Association in Birmingham, in 1849, Mr. Elkington stated, "that a few drops of the sulphuret of carbon, added to the cyanide of silver in the decomposing cell, had the property of precipitating the silver perfectly bright, instead of being granulated so dead as it is when thrown down from the solutions ordinarily employed."

107. Should the silver deposit present a whitish surface, streaked with perpendicular *black* lines, it may be regarded as an indication that the action is attended with a development of hydrogen: this must be prevented by some of the means so often mentioned (§ 78, &c.) By careful attention at the commencement of the process the right degree of action is readily obtained; and if the process is continued (with occasional watching) for about half an hour, the medal will be beautifully coated with *dead* silver. In that condition it may remain, after being washed, and dried in blotting-paper. Or, if a burnish is desired, the leather and plate brush must be used; or it may be thrown down bright as above (§ 106.)

Mr. Bain has patented an instrument which he styles a "Voltaic Governor." The plates of the voltaic arrangement are immersed to a depth sufficient to produce the electricity required. They are suspended in the liquid as weights to a clock-work arrangement. When the action diminishes, a keeper from an electro-magnet, through which the current passes, is moved, and the plates are said to sink until enough of electricity is generated to cause the electro-magnet again to attract the keeper.†

* This principle, so often alluded to, of retarding or restraining the energy of the action, is regarded in the employment of *thin* wire; it is a very valuable adjunct to the other means (§ 78) of obtaining the same end; and may often be adopted with advantage.

† *Vide* Mech. Mag. 5th Aug. 1843.

If, instead of plating medals the object is to deposit silver in a mould, as mentioned elsewhere, the same preparations are to be made; but the mould should be allowed to remain for some minutes (more or less according to the thickness required) subject to the action of the current. It may then be removed, and after being washed with water, and afterwards with water containing a few drops of nitric acid, may be placed with proper connections in a copper solution (§ 57,) to remain there till it is sufficiently backed up with this metal.

108. *Gilding by means of a single Cell.*—The operation of gilding is conducted much in the same manner as that of plating—gilding, however, requiring a little longer time, and occasionally hot solutions.

109. The operations of gilding and plating seem at first to have been very generally affected by means of the single cell, in a manner more or less in accordance with the directions I have just given, as the nature of the case permitted. In fact, *plating* by this process had been adopted on a scale of some magnitude in the great manufacturing town of England; the strength of the solution being maintained by fresh supplies of the oxyd of either gold or silver. And if attention be paid to the instructions given, there is little fear of failing.

110. Before describing a method which appears far superior to this, I would direct attention to the source whence the silver and gold in the single cell process are obtained, viz. from the *oxyds*, for instance. For every ounce of these metals deposited, a quantity of the oxyd must be furnished which shall contain in it an ounce of *pure* metal; and hence for every ounce of *metal*, much *more* than an ounce of *oxyd* is consumed. The time and trouble required to effect the combination between these metals and oxygen are by no means inconsiderable; and hence the expense of first producing the oxyd of gold or silver, and then releasing either from the after-combination with cyanogen, far exceeds the actual cost of the metal employed; *how* far depends upon circumstances. The object, however, may be accomplished with far more certainty, and at considerably less expense by means of an additional cell (§ 56,) and a plate or wire, &c. of gold or silver, to keep up the strength of the solution, as in the case of sulphate of copper. This method is now adopted generally by several patentees; for experiments with solutions of silver and gold in union with cyanogen, have shown that *cyanogen nascent at the positive plate in a decomposition cell will combine with silver and also with gold.* This furnishes a means of gilding and plating, by the use of a generating cell to furnish the electricity, and a decomposition cell to contain the cyanide solution; the nature of the changes produced has already (§ 95) been described.

111. *Battery Process for Plating and Gilding.*—The generating cell for acting upon solutions of silver need not be large. A pint Daniell, similar to that in the wood-cut, or a series of

FIG. 13.

two, is sufficient for larger medals than can be placed in the decomposition cell attached. The latter is of porcelain or glass. Of course, the size varies according to the extent of the experiment. The zinc may be used unamalgamated, and excited with salt and water; the copper cell of the Daniell's battery contains, as usual, a solution of the sulphate (§ 57.) Gilding may be better accomplished by using three cells of Daniell's battery.

112. *Voltaic Condenser.*—Prof. De la Rive has introduced an instrument, which he has named the *Voltaic Condenser,**

* Vide *Arch de l'Elect.* No. 8, p. 173, and *Elec. Mag.* p. 38.

and which may probably be of some service in electro-gilding and plating. Its property is to give to one cell of a battery the intensity of two or three, being the power required for these processes; and it does this at the expense of only *one* equivalent of zinc. It is well known to electricians that at the moment contact is made with the battery, so as to send a voltaic current along a wire in one direction, a *secondary current*, which endures but for an instant, is induced in the wire in the reverse direction; and when contact is broken, so that the original current ceases, the secondary current is induced to move in the direction contrary to its original motion; and therefore in the *same* direction as that pursued by the primary current, when contact was first made. The intensity of this current greatly depends on the quantity, the character, and the form of the wire employed; and if the wire is coated with silk and wound round a bobbin, the intensity is greatly increased. M. De la Rive uses 100 convolutions of three stout copper wires, and places within the coil a bar of soft iron, the use of which will soon become evident. The object of the arrangement is to convey the battery current, and with it the secondary current through the solution to be decomposed.

113. For example, we will select the gold solution to illustrate the use of the *condenser.* Metallic connections are applied between the ends of the coil and the two terminations of a Daniell's or Smee's battery. The connections are continued to a vessel containing the gold solution, the arrangement being somewhat like the figure ∞, where the generating cell is to the right, the coil in the centre, and the decomposition cell to the left. The current, on leaving the battery, has thus the choice of two paths, the one being through the coil, the other through the solution; but from the great comparative resistance of liquids, compared with metals, far the larger portion would pass through the coil, while a comparatively small share would traverse the solution of gold. In passing through the coil, however, it converts the soft iron core into a magnet; this magnet instantly attracts a piece of iron, which is so arranged that on being raised, it removes a wire and thus breaks off communication between the coil and the generating cell, except by means of the cell containing the solution. The current, therefore, now passes through the gold solution. But when the coil ceased to be alone in the circuit, a secondary current was induced in the same direction as the original battery current; this, therefore, joins with the said generating current, and *both pass together* through the gold solution; by which means the actual power of the battery is very greatly exalted. Now, the iron core loses most of its magnetism, as soon as the liquid is included in the circuit; and hence the piece of iron, the raising of which broke contact, falls again, and the coil is again included, when the same phenomena recur; and thus, by a continual succession of breaking and making contact, the *current of a moment*, namely, the *secondary current*, is created, and employed with very great advantages. My readers must be content with this general description; and I must trust to their own ingenuity for making arrangements agreeable to these directions.

114. *Application of Heat.*—Considerable advantage accrues in all cases of the deposition of metals where adhesion is desired, by the use of heat. It expands the baser metal, and so far opens its pores that the subsequent contraction, consequent on the effect of common temperatures, is likely to operate favorably in binding the metals together. It has other advantages, especially in gilding. The mode of heating the solutions will depend entirely on the circumstances under which the experiments are conducted. If a hot stove, or a sand-bath be at hand, the object is soon accomplished; but, in most cases, a simple plan is to use a lamp and a glass or other retort, and convey steam by a glass tube into the metallic solution, either of the single cell apparatus, or that contained in the decomposition cell.

115. With regard to the time requisite for plating and gilding, it is entirely dependent on the nature and uses of the article. The thickness of the deposit, of course, depends on the duration of the action. For medals, and such things as are not exposed to wear, a few minutes' immersion may be enough; for spoons, forks, plated goods, &c., subject to much wear, six or eight, or even more hours; always taking care to watch the process at times, in order to prevent the occurrence of the black lines; whenever they appear, the action must be retarded. Large objects, or those which are subject to a long action, should be occasionally withdrawn, and their position should be altered; so that a uniformity of deposit may occur. *Motion* of the articles during the process has been recommended, and with some show of reason. The readiest method of producing it is to suspend the article in the solution from a common bottle-jack, and connect the latter with the battery. Or, on the large scale, when it would not be convenient to have a roasting-jack for each group of articles, it might be convenient to have a constant flow of the solution. The surface obtained in the deposition of silver by electrolysis is technically termed "dead." Medals thus coated, if care be exercised during the operation, are very beautiful, and should be prepared for the cabinet by simply washing in water. If a bright surface is desired, they are polished with a leather and plate powder (§ 107.) Ordinary plated goods are finished off by polishing and burnishing. A steel or agate burnisher is used. In articles of jewelry some parts are left dead, and others are made bright.

116. *Preparing Surfaces to unite with Gold and Silver.*—But we are going on too fast; I must return to certain things preliminary to plating and gilding, which I had passed over, in order not to interrupt the progressive illustrations of the nature and preparation of the solutions. I allude to the preparation of the surfaces, previous to applying the metals; which is a point of such paramount importance that, unless duly regarded, all subsequent operations will be futile; and it would be vain to hope for perfect adhesion between the metallic base and the deposit; the latter will rise up in blisters where the surface is not properly prepared, and can easily be rubbed off.

117. There are two methods of preparing metals for the reception of other metals—the wet way, and the dry way. The experiments of M. Becquerel and others are decidedly in favor of the latter; but, as it cannot be adopted, except in certain cases, where the work of the article is plain, and the article itself is not delicate, it will be necessary to describe both modes. The main intent of cleansing is that the contact between the two metals may be perfect; and it effects this by removing grease and all extraneous matter, especially the oxyds, which are ever found on the surface of the less noble metals.

118. *Cleansing by the Dry Method.*—The advantage of the dry process over any in which moisture has been employed, is that, in the latter case, several seconds, at least, must always pass between the act of removing the article from its last liquid bath, and placing it in the solution of the metal to be deposited; and during this short interval, the article, or some portion of it, very frequently undergoes an alteration, trivial, indeed, but still an alteration, by the action of the air, which produces a film of oxyd, infinitely thin, it is true, yet quite enough to militate against the success of the experiment, as regards permanent adhesion. Therefore, wherever the dry process can be adopted, it is decidedly the better; although, from the very nature of the articles subject to the process, the number of cases in which it is available is very limited. The dry process is merely the operation of scouring with sand, or glass, or emery-paper, as the case may be, or with very fine powder of pumice-stone; using clean brushes, utterly free from grease. Sometimes fine files may be used; indeed, all depends on the value and character of the article operated upon. It must be remembered throughout that grease and oxyd are the great enemies to be expelled; and therefore, especial care must be taken to avoid contact with the moisture of the hand, which is of a nature to produce either.

119. *Cleansing by the Wet Method.*—The solutions employed may be divided generally into two classes, the acid and the alkaline; the action of the former is directed more towards the removal of oxyds, &c.: that of the latter to the removal of grease. As a rule, I would always follow the use of an acid bath by an alkaline, having first washed away the acid in several waters; and this may be done, whether the operation commence with an alkaline bath or not. The following are some of the modes in use; they are all effectual according to the circumstances which give preference to one over the other: The method recommended by M. Boettiger, in his account of gilding, given in the *Annalen der Chimie und der Pharmecie*,* may be adopted. He says: "It is very necessary to rub the metal according to circumstances,† with extremely fine sand, moistened with hydrochloric acid mixed with a little chalk, so that there shall remain no trace of oxyd of copper." Another effectual method is immersing the article in a mixture technically termed "pickle." This may be made of

| | |
|---|---|
| Sulphuric acid, . . . . . . | 64 parts |
| Water, . . . . . . . | 64 " |
| Nitric acid, . . . . . . | 32 " |
| Muriatic, . . . . . . . | 1 part. |

The "pickle" is used by tying a wire round the article and immersing it for a second or two; the action is very energetic, and, of course, is not suited to the preparation of medals: for medals, the mixture should be very much diluted, and they should remain in it for a short time. A mere bath of dilute nitric acid is often used. Nitric acid, mixed with sea-salt and soot, is often rubbed on the article. Concentrated sulphuric acid and sea-salt is another mode. Of the alkaline solutions are caustic soda, or solution of soda and ammonia, or caustic soda and sal ammoniac; or the articles may be boiled in a solution of common soda or potash, which is a very good method of cleaning them.

120. Whatever solution is used, whether acid or alkaline, or the detergent paste of soot, or chalk and acid fresh water must not be spared for rinsing off all remaining traces; and the article must be dried for immediate use by pouring over it *boiling* distilled or rain water; or, if the process of deposition is not to be commenced immediately after the rinsing, it may be buried in hot or cold boxwood sawdust, until required; it may often be dried for immediate use in hot saw dust. In addition to the detergent methods already given, an ancillary

* Vol. xxxv. p. 350.

† *I. e.* when it can be done without injury to the object of experiment; and this, too, must be the guide in the application of the other modes.

12*

means, which has been found effectual, depends upon the fact that metallic and other surfaces, after exposure to the air for some hours, become coated with a film of air so intimately as to retain it, even (as in electrotype cases) between themselves and any metal deposited upon them. In fact we have been advised, in copying large subjects by electrotype, to take advantage of this, and to allow the film to arrange itself, before the plate is submitted to the action of the battery. For it is found that the presence of this natural film very materially operates in preventing adhesion between the plates and the deposit: whereas, in the absence of the film, unless its place has been supplied by something else, other things being in order, the *two* will effectually become *one.* We are advised, too, after soldering a wire to a copper plate, to allow the latter to remain an entire day, to regain the film of air which had been driven off by the heat. Carrying out this principle, the boiling alkaline solution and the boiling water answer a double end; and hence are very effectual means of promoting perfect union between the metals. Heat operates still more favorably in causing the expansion of the metal, as I mentioned when recommending its adoption in the process itself of electric deposition. Iron may be prepared and cleaned by electrolytic action, as described elsewhere (§ 166.) In preparing steel for gilding it must be polished without oil, as the oily particles adhere so closely that it is scarcely attacked by strong muriatic acid. The last cleansing method I have seen, and it is a capital one, is to scour the surface with Calais sand, moistened by the silver or gold solution, and rubbed in with a scratch-brush.

121. *Amalgamation to promote Adhesion.*—Another method in this preparatory stage of the proceedings, to which I shall allude, is that recommended by M. Becquerel;* and which promises to be of great avail in insuring a successful termination to the experiment. After the articles are thoroughly cleaned, according to the instructions now laid down, they are dipped into a solution of proto-nitrate of mercury; when taken

* *Vide* Les Comptes Rendus, July 3, 1843.

out they are washed in abundance of water; and are then rubbed with leather, in order to promote the equal spread of the mercury. These operations are repeated until the whole surface is well coated with mercury. The ultimate character of the metallic deposit depends on the surface given to the mercury; if the employment of the leather is only such as is needed to effect the more equal diffusion of the mercury, the surface is dull or dead, and so is the deposit; whereas, if brisk friction is applied, and the mercury receives a good polish, such will be the character of the metal thrown down. And thus may burnished gold or dead gold be produced at pleasure. By adopting this method of giving a mercurial coat as the foundation for the plating or gilding (and it is especially valuable for the latter,) a double advantage accrues; the close adherance between the metals is insured—and a coating of gold of any thickness may be thrown down. The mercury is subsequently driven off by heat; either heat applied for the purpose, or the heat employed in some of the operations by which the work is finished.

122. German silver is prepared by allowing it to remain for three or four hours in a cold solution of carbonate of potash. It is then washed in cold water, and dipped into dilute nitric acid. Again washing and drying it, it is rubbed with leather; and immediately before placing it in the silver solution it is dipped into a solution of common salt, containing a little gum.

123. *Cleaning Electro-plate.*—Electro-plating, especially of dead silver, is very liable to turn yellow, after a few day's exposure to the light. M. Mourey found* that this was due to the decomposition of a cyanuret or a sub-cyanuret remaining on the silver surface on its emersion from the solution. He removes it in the following manner. The articles are covered with a thick layer of dissolved borax, and, being placed in a muffle, are submitted to a heat somewhat below cherry-red, which is sufficient to calcine the borax. They are then thrown into water acidulated with sulphuric acid, and allowed to remain.

* *Vide* Comptes Rendus, April 3, 1843, p. 660.

After being withdrawn from this, they are washed in water and dried, first in hot sawdust and then on a stove or otherwise. The result is the production of that white color so essentially requisite to dead silver, especially in articles of jewelry. I may add to this a process for cleaning tarnished silver in general, which, though not much known here, is practiced constantly by the natives in India. A few tamarinds are placed in water contained in an earthern vessel, and the silver articles are boiled in it for a time, and they emerge clean and very white.

124. *Gilding-Wax.*—The proper color is given to the surface of electro-gilding by covering it with *gilding-wax*, and heating it till the mass begins to smoke. Gilding-wax consists of the powders of saltpetre, sal ammoniac, sulphate of iron, and verdigris, mixed with melted wax. This operation removes the brassy appearance, which the surface often presents, and gives the rich gold color, on which the beauty of the work depends.

## Figures and Tables

**Fig. 11. f1:**
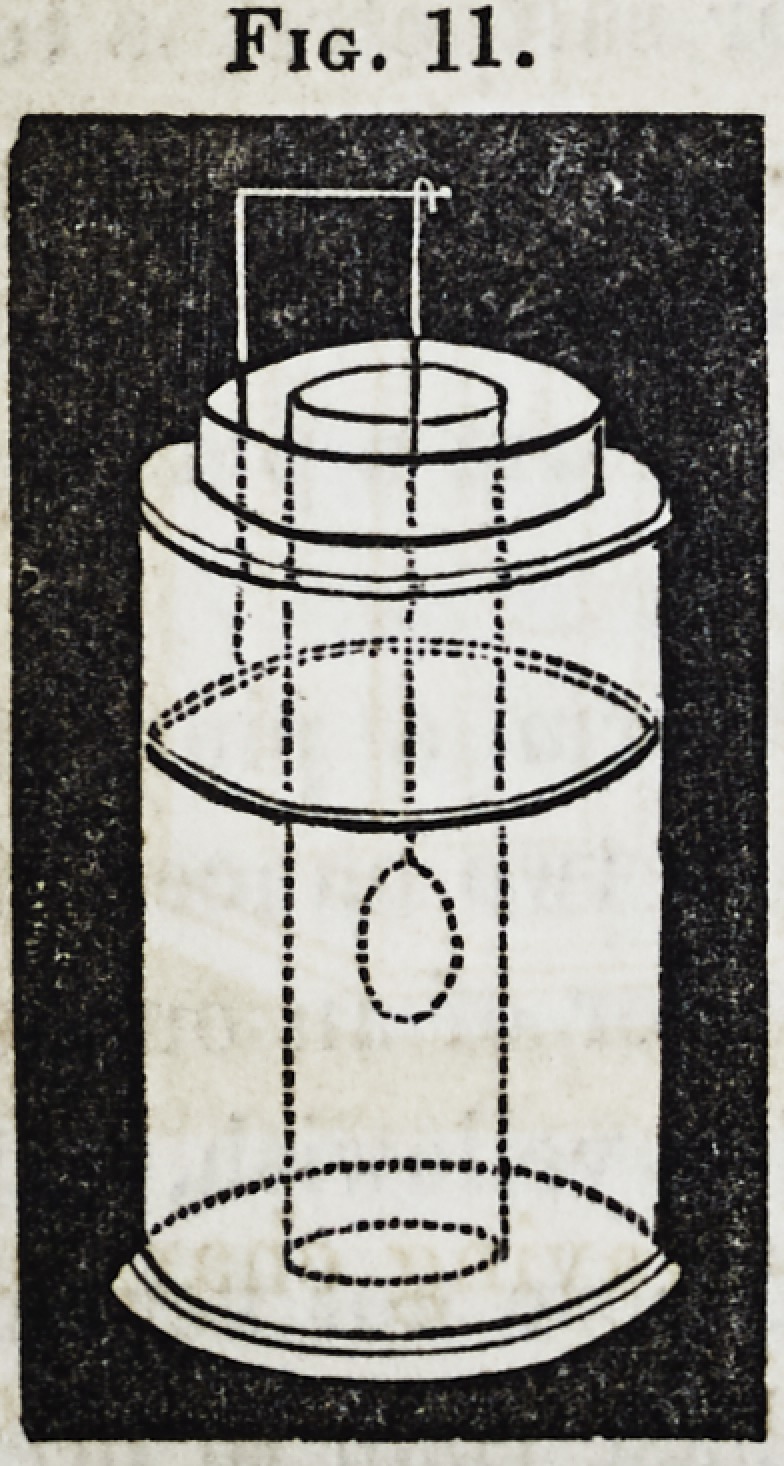


**Fig. 12. f2:**
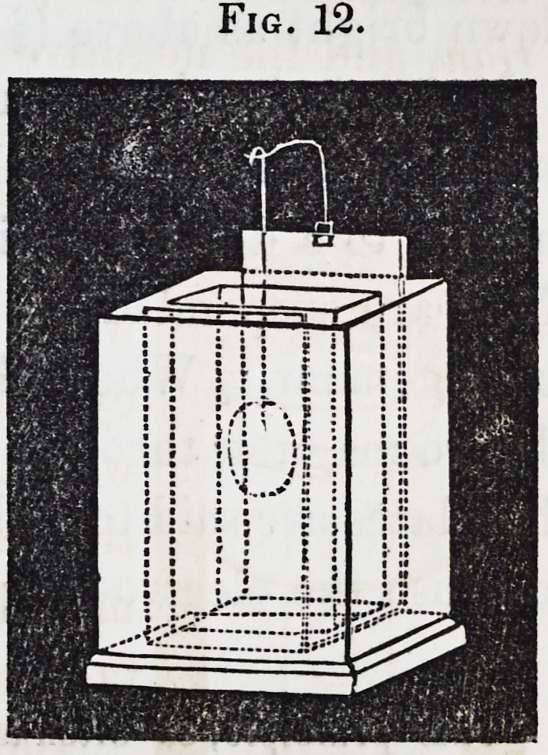


**Fig. 13. f3:**